# Does One Health need an ontological turn?

**DOI:** 10.1080/09581596.2025.2497358

**Published:** 2025-05-07

**Authors:** Andrea Kaiser-Grolimund, Salome A. Bukachi, Julia Karuga, Laura Kämpfen, Frédéric Keck, Jakob Zinsstag, Hannah Brown

**Affiliations:** ^a^ Department of Epidemiology and Public Health, Swiss Tropical and Public Health Institute, Basel, Switzerland; ^b^ Department of Public Health, University of Basel, Basel, Switzerland; ^c^ Department of Anthropology, Durham University, Durham, UK; ^d^ Department of Anthropology, Gender and African Studies, University of Nairobi, Nairobi, Kenya; ^e^ Department of Social Sciences, University of Basel, Basel, Switzerland; ^f^ Laboratoire d’Anthropologie Sociale, Paris, France

**Keywords:** One Health, ontology, anthropology, human-animal health, multispecies ethnography

## Abstract

One Health has gained global prominence in recent years. Alongside its emergence, there have been extensive social science critiques. In this contribution, we make the case for the value of recent theoretical discussions in the field of anthropology – sometimes referred to as an ‘ontological turn’. We argue that taking theory seriously benefits One Health as an integrated approach that has interdisciplinary collaborations at its heart, but which encounters challenges when conversations based on different epistemological and ontological positions result in voices talking past each other. In this contribution, we offer two examples of what One Health specialists can gain from anthropologically-informed ontological thinking. Both require questioning ontological premises. Firstly, questioning assumptions about distinctions between animals and humans. Secondly, questioning the universality of biomedical knowledge. In the conclusion, we underline the importance of an ontological openness when it comes to the constitution and position of the actors as well as different bodies of knowledge that are involved in One Health and we show that talking to each other with awareness of different ontological positions is not impossible.

## Introduction

As One Health, Planetary Health and related approaches gain influence, interdisciplinary approaches to health problems are in the foreground. Often, the contribution of anthropology and other social sciences is assumed to be primarily an empirical one, to enrich understandings of the socio-economic and political determinants of health as they affect multi-species communities. This commentary takes a different approach. We make the case for the value of recent theoretical discussions in anthropology which have made multispecies relations a core focus for the discipline and endeavored to take seriously communities who see animals as social actors. We argue that these theoretical insights – sometimes referred to as an ‘ontological turn’ – can benefit One Health as an integrated approach that has interdisciplinary and transdisciplinary collaborations at its heart, but which encounters challenges when conversations based on different epistemological and ontological positions result in misunderstandings. After briefly outlining the content of the ‘ontological turn’ in anthropology, we offer two examples of anthropologically-informed ontological thinking with relevance for One Health specialists. Both require questioning ontological premises. Firstly, questioning assumptions about distinctions between animals and humans. Secondly, questioning the universality of biomedical knowledge. We argue that these ontologically-informed approaches offer an useful tool to develop more just and sustainable One Health solutions.

## Background

‘One Health’ has gained prominence in recent years following global crises including the H1N1 swine influenza and Covid-19 pandemics, and the recent adoption of a shared definition of One Health by high-level global institutions as an ‘integrated, unifying approach that aims to sustainably balance and optimize the health of people, animals and ecosystems’ (Adisasmito et al., 2022, p. 2).

There have been extensive social science critiques of One Health alongside its emergence. In a recent volume edited by Irus Braverman, the author calls for a critical orientation towards ‘more-than-One’ Health, suggesting a ‘more-than-human’, ‘more-than-zoonotic’ and ‘more-than-science’ approach to One Health (Braverman, 2022, p. 2). These reflections take up some of the earlier criticisms about the often marginalized role and the potential contributions of social science (or anthropology) in One Health (e.g., Steffens & Finnis, 2022; Whittaker et al., 2021; Woldehanna & Zimicki, 2015; Wolf, 2015), and have been accompanied by calls for its decolonization (e.g., Davis & Sharp, 2020; Lainé & Morand, 2020).

At the core of much criticism is the idea of ‘One-ness’ embedded in the approach and the implications that this universalizing claim entails. With reference to John Law, Steve Hinchliffe points to the problems related to a ‘one-world-ist ontology’ of One Health (Hinchliffe, 2015, p. 34). According to Law, ‘one-world metaphysics are catastrophic in North-South encounters’ as such metaphysics ‘reduce difference’ (Law, 2015, p. 134, see also Hinchliffe, 2015, p. 31). Critics argue the well-intentioned idea of one shared world often overlooks not only historical inequalities but also the fact that communities have different ways of living with other beings, and that ‘health’ is not the same thing for all humans and non-humans, everywhere in the world.

Much of this critique is underpinned by ontological arguments. There is not one common definition of ontology or the ‘ontological turn’ in anthropology. One way to describe it, is that ontologically informed anthropology is concerned with studying reality (Kohn, 2015). Such an approach raises methodological and theoretical questions and is often related to posthumanist concerns, when the ‘reality’ that it studies ‘encompasses but is not limited to humanly constructed worlds’ (Kohn, 2015, p. 312, see also p. 313). A key insight of ontological approaches is that communities not only have *different representations* of the world, but may differ about what *counts as* a ‘world’ and over the categories of ‘human’, ‘animal’, or ‘environment’, which may either not exist or not in ways commensurate with the categories of Euro-American science and philosophy, often perceived as the gold standard by which other perspectives are measured (c.f., Heywood, 2017/2023; Kohn, 2015). Hence, such approaches turn questions on ‘*how one sees things*’ into questions of ‘*what there is* to be seen’ (emphasis in the original, Holbraad & Pedersen, 2017, p. 5).

Early One Health literature argued that ‘[s]imilarly to the human-human relationship, the human-animal relationship is governed […] by culture and religion’ (Zinsstag et al., 2015, p. 18). This is an important insight, but ontological interventions in anthropology go further than arguing that human-animal relationships are shaped by cultural norms, to question how far the concept of ‘the human-animal relationship’ functions as a category that can be employed cross culturally. For example, Brazilian anthropologist Eduardo Viveiros de Castro (1998) challenged Euro-American assumptions about the valence of the ideas of what is ‘natural’ and given and ‘cultural’ and human-made, through research in the Amazon with people who described both humans and animals as ‘subjects’ who recognized each other as ‘persons’. Building partly on his ethnographic research in the same region, French anthropologist Philippe Descola described four distinct ontological modes found in different world regions, labelling the ontological assumptions of the modern West, including Biomedicine, which assume a shared natural world that can be acted on by humans, ‘naturalism’ (Descola, 2013). These analyses join a vast literature in the social sciences that has shown that Euro-American ideas about what constitutes ‘humans’ and ‘animals’, and the ways they relate to each other, are not universal.

However, these ontologically informed approaches are not without controversy. Scholars, and especially fellow anthropologists, have critiqued the way that such approaches risk creating the impression that people live in different, incommensurable ‘worlds’, leading to a form of hard relativism, overlooking historically shaped realities and political concerns relevant to the studied communities (c.f., Bessire & Bond, 2014; Kohn, 2015). Nonetheless, taken carefully, the concept of ontological difference offers possibilities for understanding radically different ways of being in, and knowing, the world. We argue that an awareness of ontological distinctions can help One Health experts to navigate the many forms of difference encountered in an endeavor that is based on the idea that there is value in developing collaborative and integrated approaches to health. One Health is founded on understandings of difference, and the need to integrate differences. But what kind of difference? And how and when should this integration happen? Building on the above mentioned social science criticism and on scholarship who has argued for advancing One Health with (critical) posthumanist approaches and ontologically informed thinking (e.g., Collins, 2023; Rock, 2017; Van Patter et al., 2023), in what follows we offer two brief examples that show how taking ontological difference seriously, and attempting – as anthropologists do – to understand different ontological positions in their own terms, would be a beneficial move for One Health.

## Humans and animals

The prevailing academic, legal and governance systems of most countries in the world clearly separate between humans and animals. These separations have grown historically, for example, the early universities had only medical faculties and included much later veterinary medicine (Zinsstag et al., 2005). One Health approaches frequently perpetuate separations between animals and humans despite attempts to think across them. As some critical scholarship argues, these separations are necessary to justify the need to bring these distinct communities together, sharing one world and consequently one health (Keck & Lynteris, 2018, p. 25). Often, these separations bracket the world into distinct forms of life aligned to particular forms of expertise (animal: veterinarian vs. human: biomedicine vs. culture: social science). Such framings not only problematically situate scientific knowledge outside of culture, but they may also have ethical consequences as they incorporate implicit value judgements and hierarchies. For example, in pandemic contexts One Health approaches often prioritize the (physical) health of humans over the health of other beings, recommending measures such as culling of animals, in order to prevent a zoonotic disease from spilling over to humans, whereas the potential risks of human practices for animals and the environment might be ignored. In such framings, it is assumed that animals that threaten or harm human health can – and should – be extracted from human worlds whereas other ways of thinking about the risk posed by animals, or their value, can be overlooked. While in the literature, the connection between an ontological lens and human-animal ethics is not straightforward, ethnographic explorations have shown that value judgements and hierarchies within multispecies communities may vary and may have political implications (Law, 2010).

Where ‘One Health’ often focuses on animals as hosts or vectors of diseases, recent work has explored animals and microbes as actors with whom we share affective, economic, and political forms of coexistence (e.g., Haraway, 2008; Latour, 1988; Nading, 2013). Such work not only offers rich empirical insights into the complex ways humans are entangled in multispecies relations (Kirksey & Helmreich, 2010). It also enables us to question implicit assumptions about what it means to be ‘human’ or ‘animal’ and how these assumptions might limit our capacity to understand multispecies health. Our interdisciplinary engagements with One Health practitioners suggest that they too appreciate the value of questioning ontological assumptions. For example, social scientists have asked what it would mean, in the case of malaria control, to think of humans, infrastructures, mosquitoes and environments all as ‘actors’ (Mitchell, 2003), transforming understandings of agency into a more-than-human quality. The relevance of questioning ontological assumptions can be highlighted by one of our discussions with an interdisciplinary group of One Health specialists about the ‘ontological turn’ in anthropology when an epidemiologist asked skeptically where it would lead if even a tiny mosquito were considered an actor. While the anthropologist who gave the input began a lengthy response, another veterinarian in the room interrupted. He responded simply that, in his opinion, a mosquito should very well be considered an actor because this tiny little creature can kill you.

## De-hierarchising knowledge(s)

Insights into the complex, multidimensional ways people relate to animals can also provide valuable understandings for One Health practitioners by challenging underlying assumptions of biomedical knowledge. Taking ontology into account is a way to take local realities seriously and to challenge hierarchies of knowledge. This goes beyond the question of whether people use the same categories to describe experience. Exploring co-existing bodies of knowledge – as equally valuable even while potentially incommensurate – trains attention to diverse experiences that matter for the way health is understood.

Most One Health experts are trained in biomedical or natural sciences. This means that their assumptions about the world are based on verifiable ‘facts’, driven by a particular body of (biomedical) knowledge. Such knowledge usually presumes that ‘the social world can be understood like the natural world’ (Tompson et al., 2021, p. 3). Although there are increasing examples of attempts at non-hierarchical, collaborative, and transdisciplinary approaches that take up ontological concerns (Zinsstag et al., 2023), many One Health projects consider ‘local’ or ‘lay’ knowledge as an obstacle, or dismiss certain ontologies as ‘belief’ rather than knowledge. For example, One Health approaches to antibiotic use in farming often talk about ‘irrational’ use of medicines in a way that implicitly blames certain people – usually poor farmers in the Global South – for what are seen as ill-conceived behaviours (Denyer Willis et al., 2023). Similarly, in the aftermath of the Covid19 pandemic, consumer preferences in China aligned values of quality and freshness with live chicken purchased from a ‘wet market’, rather than meat that had been refrigerated. Such practices were viewed with skepticism in the global community (Lynteris & Fearnley, 2020).

Embracing respect for different ontological positions within One Health requires understanding different interpretations of (co-)existence and knowledge, especially those of communities who are targeted by interventions. Victoria Brookes et al. (2020) for example, explored the management of dogs in remote communities in northern Australia that valued the family-oriented roaming of dogs and consequently conflicted with expectations around dog keeping held by the Australian government. The authors therefore emphasize the need to acknowledge different modes of dog ownership and to consider different forms of community-based management as well as locally grounded perceptions on human-animal encounters as the foundation of One Health interventions (Brookes et al., 2020). Often, people who share their livelihoods with animals already incorporate aspects of ‘lay One Health’ (Mwangi, 2022) that can be drawn upon in developing appropriate interventions (see also MacGregor & Waldman, 2017; Mburu & Heitz-Tokpa, 2024). A focus on the ontological commitments that underpin the way a community understands their relationships with animals could change how we understand disease and health between species, repositioning complexity not as a problem to be overcome, but as an intellectual resource that improves our understanding of relevant context, and also suggests avenues of working together for shared health.

## Conclusion: meaningful entanglements

Ontologically informed questions ask One Health experts to turn the spotlight onto themselves and consider the underlying logics of their assumptions. In social science, we call this approach to knowledge production ‘critique’. We argue that it is social scientists’ experience of critique – i.e. of challenging taken for granted knowledge settlements – that is the key contribution that social scientists bring to One Health, not simply their expertise in accessing certain kinds of empirical material (McLeish & Strang, 2014). Questioning ontological settlements does not mean that we are forced to concede that people live in different worlds and that communication across different ontological positions is impossible. An ‘ontological turn’ for One Health, in our view, is instead a theoretical exercise that is aimed at critiquing one’s own assumptions and knowledge practices, underpinned by a commitment to consider – and value – different ontological positions. The One Health field consists of a quickly growing and heterogeneous community of experts including many who are aware of such ontological considerations. How such considerations – that go deeper than a mere openness to a diversity of views – can be reflected in One Health practices on the ground needs further exploration in order to underline its broader potential.

This paper has outlined a ‘productive complication’ of One Health by addressing the critique of the ‘One-ness’ on ontological grounds. One Health is already a very complex endeavor. But if the very aim of One Health involves sharing ideas and listening to different voices within the human-animal-environment-plant-nexus, then it is crucial to have an ontological openness when it comes to the constitution and position of the human and non-human actors as well as different bodies of knowledge that are involved.

## Data Availability

Data sharing is not applicable to this article as no new data were created or analyzed in this study.
